# Clinical Features, Incidence and Treatment Outcome in Pregnancy-Associated Osteoporosis: A Single-Centre Experience over Two Decades

**DOI:** 10.1007/s00223-023-01139-3

**Published:** 2023-10-11

**Authors:** Elizabeth Orhadje, Kathryn Berg, Barbara Hauser, Stuart H. Ralston

**Affiliations:** Centre for Genomic and Experimental Medicine, Institute of Genetics and Cancer, University of Edinburgh, Western General Hospital, Edinburgh, UK

**Keywords:** Osteoporosis, Pregnancy, Lactation, Fracture

## Abstract

Pregnancy-associated osteoporosis (PAO) is a rare syndrome which typically presents with vertebral fractures during pregnancy or lactation. The medical records of sixteen patients with PAO who presented to a specialist clinic at the Western General Hospital in Edinburgh over a 20-year period were reviewed to evaluate the mode of presentation, potential risk factors and response to treatment. The most common presentation was back pain occurring in 13/16 (81.2%) individuals due to multiple vertebral fractures. The diagnosis was usually made postpartum and in 12/16 individuals (75.0%), PAO presented during the woman’s first pregnancy. Medicines which could have contributed to the development of PAO included thromboprophylaxis therapies in 8 subjects (50.0%), inhaled or injected corticosteroids in 5 (31.3%), anticonvulsants in 2 (12.5%) and a LHRH agonist in 1 (6.3%). Five individuals reported a family history of osteoporosis, and two pregnancies were complicated by hyperemesis gravidarum. Treatments administered included calcium and vitamin D supplements, bisphosphonates and teriparatide. Bone mineral density increased following the diagnosis in all cases, regardless of treatment given. One patient had further fracture during follow-up, but four patients had subsequent pregnancies without fractures. We estimated that in this locality, the incidence of PAO was 6.8/100,000 pregnancies with a point prevalence of 4.1 per 100,000 women. This case series indicates the importance of family history of osteoporosis and thromboprophylaxis drugs as risk factors for PAO while also demonstrating that the reductions in bone density tend to reverse with time, irrespective of the treatment given.

**Supplementary Information:**

The online version contains supplementary material available at 10.1007/s00223-023-01139-3.

## Introduction

Osteoporosis is a common condition which predominantly affects older people. However, it can also affect pregnant or lactating women, where it is known as pregnancy-associated osteoporosis (PAO) [1, 2]. This is a rare syndrome which is said to affect every four to eight per million pregnancies [3], but there have been no good epidemiological studies in which its true incidence or prevalence has been assessed. Over 300 cases of PAO have previously been reported in the literature [4]. Patients with PAO typically present in the third trimester of a first pregnancy or the early postpartum period with multiple vertebral fractures, which result in back pain, height loss and functional impairment [3, 5, 6]. It is still unclear what causes PAO, but many risk factors have been suggested. These include having a family history of osteoporosis [7] and using heparin or corticosteroids during pregnancy [6]. Calcium and vitamin D supplements [8], bisphosphonates [9] and teriparatide [10] have all been used to manage the condition empirically, but there have been no randomised controlled trials to assess the effectiveness of these approaches. The aim of this study was to analyse the clinical features and treatment outcomes of sixteen patients with PAO who were treated at a tertiary referral centre for osteoporosis and metabolic bone disease to identify any patterns that clinicians should recognise when diagnosing and managing women with the condition.

## Patients and Methods

The case series comprised sixteen patients who had been referred to a specialist clinic for bone diseases at the Western General Hospital, Edinburgh between January 2001 and December 2022. Details of each patient were held on a database of individuals with rare bone diseases including PAO who had presented to the clinic over the study period. We retrospectively reviewed electronic medical records of patients who had a diagnosis of PAO and extracted relevant demographic and clinical data. The diagnosis of PAO was made on clinical grounds by the presentation with fractures proven by skeletal imaging and/or the finding of reduced bone mineral density (BMD) during pregnancy or postpartum.

Measurements of BMD were made by dual energy X-ray absorptiometry (DEXA) using a Hologic QDR 4500 system. Relevant demographic and clinical data were obtained from review of their electronic medical records, including information about their health during pregnancy, fractures, family history of osteoporosis, past medical history and drug treatment. Statistical analysis was performed using IBM SPSS Statistics version 25. General linear model ANOVA was used to compare BMD responses to different treatment types entering age, BMI and baseline BMD into the model as explanatory factors. The correlation between duration of breastfeeding and response to treatment was examined using Spearman’s test.

## Results

### Baseline Characteristics and Clinical Features

The characteristics of the study cohort are summarised in Table 1. The average age at presentation was 34.1 years with a range of 29–40 years. Measurements of height, weight and body mass index were unremarkable. The most common presentation was with multiple vertebral fractures. The thoracolumbar vertebrae were most frequently affected, but one patient had a sacral fracture. Only one patient had non-vertebral fractures, which were multiple ankle fractures. Back pain was a presenting symptom in 81.3% but one patient presented with pain in the ankles due to fractures at this site. Other symptoms of PAO included height loss and kyphosis. Most of the women experienced PAO during their first pregnancy. There was a family history of osteoporosis in 31.3% and 12.5% had had previously suffered a fracture before the diagnosis of PAO.

**Table 1:** Clinical characteristics of study group

Demographic characteristics	
Age (years)	34.1 ± 3.0 (29 to 40)
Weight (kg)	64.9 ± 9.6 (43.4 to 78.2)
Height (cm)	157.7 ± 6.6 (149.5 to 169.0)
BMI (kg/m^2^)	26.1 ± 3.6 (19.0 to 32.1)
Smoked before pregnancy	2 (12.5%)
Clinical presentation	
Fractures	15 (93.8%)
Vertebral fractures	14 (87.5%)
Non-vertebral fractures	1 (6.3%)
Pain	15 (93.8%)
Back pain	13 (81.3%)
Pain at other site	2 (12.5%)
Height loss	2 (12.5%)
Kyphosis	2 (12.5%)
Diagnosis made during pregnancy	3 (18.8%)
Diagnosis made postpartum	13 (81.3%)
Pregnancy affected	
First	11 (68.8%)
Second	3 (18.8%)
Third	2 (12.5%)
Bone density	
Lumbar spine T-score	− 3.0 ± 1.1 (− 4.8 to − 1.1)
Femoral neck T-score	− 1.8 ± 1.0 (− 3.8 to 0.6)
Total hip T-score	− 1.5 ± 0.9 (− 3.3 to 0.6)
T-score below − 2.5 at any site	10 (62.5%)
Past history and family history	
Fracture(s) before pregnancy	2 (12.5%)
Family history of osteoporosis	5 (31.3%)
Breastfeeding	
Did not breastfeed	1 (6.3%)
Breastfed for < 3 months	1 (6.3%)
Breastfed for 3–6 months	3 (18.8%)
Breastfed for 6–12 months	3 (18.8%)
Breastfed for > 12 months	1 (6.3%)
Breastfed for unknown duration	3 (18.8%)
Breastfeeding status not recorded	4 (25.0%)
Follow up	
Went on to have further pregnancy	4 (25.0%)
Experienced further fracture	1 (6.3%)

Values for age, weight, height and T-scores are mean ± standard deviation (range), while all other values are number of patients (percentage of group)

*BMI* body mass index

The BMD values were lowest in the spine with an average T-score of − 3.0, whereas average BMD values at the femoral neck and total hip were in the osteopenic range with T-score values of − 1.8 and − 1.5, respectively. Overall, 62.5% of women had BMD values in the osteoporotic range (*T* ≤ − 2.5) at any site.

The diagnosis was usually made postpartum but in 3 patients (18.8%) the diagnosis was made during the third trimester. During follow-up, 4 women (25.0%) had a subsequent pregnancy without suffering a further fracture. One patient had a further vertebral fracture five years after the original presentation.

Clinical details of the individual patients are provided in the Supplementary Material.

### Breastfeeding

Information on breastfeeding was available in 12/16 patients. One woman did not breastfeed, but the remaining 11/12 (91.7%) breastfed for variable amounts of time. In the eight women where the duration of breastfeeding was recorded, we found no significant correlation between the duration of breastfeeding and change in spine BMD during follow-up (Pearson’s correlation = 0.141, *p* = 0.82), change in femoral neck BMD (0.175, *p* = 0.67) or change in total hip BMD (0.059, *p* = 0.88).

### Incidence and Prevalence

Of the 16 patients, 13 were resident in the catchment area of NHS Lothian, whereas the remaining 3 had been tertiary referrals from other health boards. Details of the number of births in NHS Lothian during the period 2001 to 2021 and the year of presentation of each patient are provided in Table 2. If one assumes that all patients who experienced PAO had come to medical attention and had been referred to the specialist clinic, this gives an incidence of 6.8 in every 100,000 pregnancies during that period. There were 315,600 females of working age (over 16 years) in the NHS Lothian area in 2021 [11] which would give a prevalence of PAO in NHS Lothian in women of working age of 4.1 in every 100,000 women.

**Table 2:** Presentation of PAO by year in relation to number of births in the study catchment area

Year	Number of births in NHS Lothian^a^	Case presented
2001	8331	N/A
2002	8210	1, 7
2003	8420	N/A
2004	8457	N/A
2005	8724	N/A
2006	9028	N/A
2007	9378	2
2008	9895	N/A
2009	9718	N/A
2010	9837	N/A
2011	9799	5
2012	9830	4
2013	9588	N/A
2014	9628	6
2015	9386	N/A
2016	9418	12
2017	9037	9, 10
2018	8748	14
2019	8511	15
2020	8151	8, 13
2021	8426	N/A
Total	190,520	13

^a^Data from: Table BT.5 Births by sex, year and post-April 2014 NHS Board area, 1991 to 2021 [Data file]. National Records of Scotland: Edinburgh; 2022. [accessed 23 May 2023]

### Risk Factors

Potential risk factors for osteoporosis are summarised in Table 3. Six patients had no predisposing medical conditions associated with osteoporosis. Four had been treated for asthma with inhaled corticosteroids and one had received a local corticosteroid injection because of soft tissue rheumatism. Eight had received antithrombotic therapy; in seven, low molecular weight heparin was used and in one factor Xa antagonists. Two had received anticonvulsants for epilepsy and schizophrenia, respectively. Two had experienced hyperemesis gravidarum and one had experienced an antepartum haemorrhage.

**Table 3:** Medical history of patients with PAO

Medical conditions during pregnancy	
Thromboembolic disease	3 (18.8%)
Asthma	3 (18.8%)
Endometriosis	1 (6.3%)
Chronic back pain	1 (6.3%)
Schizophrenia	1 (6.3%)
Epilepsy	1 (6.3%)
Hypothyroidism	1 (6.3%)
None	6 (37.5%)
Osteoporosis-predisposing medications ever taken	
Low molecular weight heparin	7 (43.8%)
Factor Xa antagonists	1 (6.3%)
Corticosteroids	5 (31.3%)
Inhaled	4 (25.0%)
Injected	1 (6.3%)
LHRH agonist	1 (6.3%)
Anticonvulsant	2 (12.5%)
Levothyroxine	1 (6.3%)
None	6 (37.5%)
Pregnancy complications	
Iron-deficiency anaemia	1 (6.3%)
Hyperemesis gravidarum	2 (12.5%)
Antepartum haemorrhage	1 (6.3%)
None	11 (68.8%)

Values are number of patients (percentage of group)

### Bone-Targeted Treatment

A variety of bone-targeted treatments were used, most commonly calcium and vitamin D supplements, either alone, or in combination with other agents including alendronate, zoledronic acid, risedronate and teriparatide. Table 4 summarises the response of BMD to the different categories of treatment provided between the visit where a patient was diagnosed (baseline) and their first follow-up visit. Greater percentage changes were achieved at the lumbar spine than at the femoral neck or total hip. There was no significant difference between the patients’ mean percentage changes in BMD at the lumbar spine, femoral neck or total hip based on their treatment type. It was not possible to investigate responses of lumbar spine BMD to teriparatide since spine BMD measurements could not be made due to pre-existing existing vertebral fractures.

**Table 4:** Percentage changes in BMD from baseline to first follow-up visit, according to site and treatment type received

Treatment	*N*	Site		
		Lumbar spine	Femoral neck	Total hip
Calcium/vitamin D	8	10.8 ± 8.5	4.9 ± 3.3	6.8 ± 2.8
Bisphosphonates^a^	6	14.0 ± 13.3	3.5 ± 4.7	3.2 ± 3.5
Teriparatide^b^	2	–	4.3 ± 0.5	3.4 ± 0.4

Percent change in BMD at each site are estimated marginal means ± SD adjusted for age, baseline BMD and BMI

^a^Three patients received alendronate, 2 risedronate and 1 zoledronic acid

^b^Changes in lumbar spine BMD could not be estimated in the patients treated with teriparatide as both had multiple vertebral fractures

## Discussion

The mode of presentation in this study was in keeping with previous studies which have reported that vertebral fractures are the most common type of fracture associated with PAO, and that they usually affect the thoracolumbar spine [2, 3, 12, 13]. While Hadji et al. reported a mean of 3.3 fractures per patient in their case–control study of PAO [6], eleven (68.8%) of our patients had fractures of five or more vertebrae. One patient in this series had a sacral fracture. This type of fracture has been previously described in two patients with PAO, where the use of LMWH was identified as a predisposing factor [14, 15]. Another patient had ankle fractures, which is an uncommon but probably underreported presentation of PAO [16]. Rozenbaum et al. described a patient with PAO who had fractures at the ankle, hip and knees, but this was associated with a cytomegalovirus infection [17] which did not occur in our patient with ankle fractures.

Back pain was the most common presenting complaint occurring in 81.3% of patients. Height loss and kyphosis were presenting features in two patients (12.5%). Height loss in this study was less frequent than in the study of Smith et al. where it was recorded 41.7% of cases, whereas the prevalence of kyphosis was the same as reported by Smith [18].

The average age at presentation was 34.1 years which is slightly higher than the average age at pregnancy of women in Scotland, which was 31.1 years in 2021 [19]. This is similar to the study of Tuna et al*.* where the mean age at presentation was 34.2 years [20], but lower than that reported by Kyvernitakis et al. who recorded a mean age at presentation of 39.5 years and proposed that PAO was an age-related disease [21].

Most patients were affected during their first pregnancies, which is in keeping with previous studies [13], and the diagnosis was most commonly made postpartum which again is in keeping with previous studies [6, 13].

This is the first study to estimate the incidence and prevalence of PAO. Since this is the only specialist osteoporosis clinic in NHS Lothian, we think it is likely all individuals with suspected PAO who came to medical attention will have been referred to our clinic. While it is possible that women who had experienced vertebral or other fractures during and after pregnancy did not seek medical attention, we feel this is unlikely. With these assumptions, we estimate the incidence of PAO to be 6.8/100,000 pregnancies with a population prevalence of 4.1/100,000 women. This is approximately tenfold higher than previously estimated in the literature where a figure of 4–8 per million pregnancies has been quoted [3, 6], but to our knowledge, this estimate is not based on any firm data.

Eight of the sixteen patients (50.0%) used an antithrombotic agent to prevent thromboembolic disease which in most cases was LMWH. This is in keeping with previous reports which have identified heparin as a potential risk factor for PAO [2, 6, 15]. The proportion of individuals treated with antithrombotic agents in this series is considerably higher than the 17% background rate of thromboprophylaxis during pregnancy in NHS Lothian (personal communication—Allyn Dick, Clinical Auditor, Women and Children’s Services, NHS Lothian). The mechanisms by which heparin might predispose to osteoporosis are incompletely understood [22]. Preclinical studies have shown that unfractionated heparin (UFH) and LMWH reduce bone formation and that UFH, but not LMWH, stimulates bone resorption [23]. Irie and colleagues [24] reported that heparin binds to and inhibits osteoprotegerin, and through this mechanism, stimulates RANKL-induced osteoclastic activity. The authors of this work speculated that LWMH may be less likely to inhibit OPG because of its smaller size, but this was not proven experimentally. Several clinical studies have reported associations between heparin use in pregnancy, increased bone loss and fractures [22]. There is less information on the effects of LMWH on bone but prospective studies have shown numerically greater increases in bone loss in LMWH users during pregnancy than controls, albeit with differences that were not significant [25].

Another potential risk factor is corticosteroid use [20, 26]. Five of our patients used corticosteroids during pregnancy, but four of these were inhalers for asthma and one was a local corticosteroid injection for soft tissue rheumatism. Inhaled corticosteroid use in patients with asthma has been associated with a decrease in BMD but the changes were small and not associated with fractures [27]. One patient in this series used levothyroxine to manage their hypothyroidism during pregnancy. While over-replacement with thyroxine is associated with reduced BMD, we know of no data to suggest that hypothyroidism is a risk factor for osteoporosis. Two patients received treatment with anticonvulsants during pregnancy, one for epilepsy and another for schizophrenia, while another was treated with an LHRH agonist for many years before pregnancy to manage her endometriosis. Though anticonvulsant use has previously been described as a risk factor for PAO [6] and as a risk factor for fractures in general, LHRH agonist use has not. LHRH agonists are known to decrease the BMD of women with endometriosis, but this can completely resolve after the treatment is withdrawn [28].

A family history of osteoporosis has been reported to be more common in women with PAO than healthy controls [7], and in our series, almost one third reported having a family history of osteoporosis. Some patients who present with features of PAO carry pathogenic mutations in the *COL1A1*, *COL1A2* and *LRP5* genes [26]. In keeping with this, Hardcastle et al. [5] and Smith et al. [2] each identified one patient who presented with PAO as the first manifestation of osteogenesis imperfecta. We did not conduct genetic analysis as part of the present study, but we are planning to do so in the future.

Four patients in our series had complicated pregnancies, two with hyperemesis gravidarum. In this regard, Hadji et al. found a higher prevalence of coagulation disorders, congenital malformations and pregnancy-related disorders in cases of PAO than controls [6]; however, a recent study did not find an association between hyperemesis gravidarum and osteoporosis [29].

As in previous studies [9, 30, 31], various bone-targeted treatments were given including calcium and vitamin D supplements, bisphosphonates and teriparatide. In most cases, BMD increased between the time of the original diagnosis and follow-up, but there was no significant difference in response between treatment types. It should be acknowledged that we cannot make any firm conclusions about relative efficacy of these agents since the treatment was not randomly allocated, and the numbers were small. Furthermore, the improvements observed could be due in part, to the known recovery of BMD after PAO even without bone targeted treatment [32]. The efficacy of different treatments for PAO remains a subject of debate. In one cases series, teriparatide was found to be superior to calcium and vitamin D supplements [30, 31], whereas a systematic review by Hong and Rhee reported that teriparatide and bisphosphonates were associated with similar increases in BMD in PAO and were superior to no active treatment [33].

It has been reported that almost a quarter of patients with PAO will experience a subsequent fracture, and that this risk correlates with the number of fractures a patient has at diagnosis [21]. However, this was not observed in the present series where only one patient had a further fracture, and this did not occur in the context of a further pregnancy.

The high proportion of individuals with a family history of osteoporosis raises the possibility that genetic factors may play a pathogenic role as has been suspected previously [26], but it should be acknowledged that shared environmental risk factors could also contribute to the finding of a positive family history of osteoporosis in these women.

In conclusion, this case series represents a significant addition to the literature on PAO. It represents one of the largest case series published to date with details of clinical and pharmacological risk factors, mode of presentation and response to treatment. Additionally, it is the only report we are aware of in which an evidence-based estimate of the true incidence and prevalence of PAO has been provided.

### Supplementary Information

Below is the link to the electronic supplementary material.

Supplementary file1 (DOCX 20 kb)

Supplementary file2 (XLSX 16 kb)
