# Supplementary material for: Clinical Features, Incidence and Treatment Outcome in Pregnancy-Associated Osteoporosis: A Single-Centre Experience over Two Decades
Source: Calcif Tissue Int. 2023 Oct 11;113(6):591–6. doi: 10.1007/s00223-023-01139-3 (PMC10673946; doi:10.1007/s00223-023-01139-3)
Supplement: Supplementary file 1 — Supplementary file1 (DOCX 20 kb) [file 223_2023_1139_MOESM1_ESM.docx]

**Supplementary Material – Case Histories**

**Clinical Features, incidence, and treatment outcome in Pregnancy-Associated Osteoporosis: A single centre experience over two decades**

Elizabeth Orhadje^1^, Kathryn Berg ^1^, Barbara Hauser^1^, Stuart H. Ralston^1^

^1^ Centre for Genomic and Experimental Medicine, Institute of Genetics and Cancer, University of Edinburgh, Western General Hospital, Edinburgh, UK

# **Individual case reports**

###

### **Case 1**

A 31-year-old woman was diagnosed with osteoporosis four years after her third pregnancy, but there was no clear history of pain or fractures during the pregnancy. She had previously experienced a metatarsal fracture several years previously. A DEXA scan was performed six months after her third pregnancy, which showed T-scores of -4.10 at the lumbar spine, -1.70 at the femoral neck and -1.90 at the total hip. She had no family history of osteoporosis, but she received low molecular weight heparin (LMWH) as thromboprophylaxis throughout her third pregnancy, because of deep vein thrombosis during her second pregnancy. Her third pregnancy was complicated by severe iron deficiency anaemia, but she breastfed her third baby for at least six months. She did not receive any osteoporosis treatment at the time of the initial diagnosis. Thirteen years after her third pregnancy, her BMD improved such that the T-score increased to -3.20 at the lumbar spine, -1.90 at the femoral neck and -0.90 at the total hip. However, when she was scanned again eighteen years after her third pregnancy, her T-score at the spine had decreased to -3.80. No hip measurements were reported this time around, as optimal positioning was not possible. Because of the continued osteoporosis at the spine, she started taking calcium and vitamin D supplements and received three intravenous infusions of zoledronic acid (ZA) at yearly intervals, nineteen years after her third pregnancy. Despite the very low BMD, the patient had no clinical fractures during a twenty-one-year follow-up period.

### **Case 2**

A 32-year-old woman presented with back pain one month after her first pregnancy and was subsequently found to have multiple vertebral fractures at T6, T8, T10, T11, T12 and L2 by X-ray. A DEXA scan performed eight months after this pregnancy showed T-scores of -3.50 at the lumbar spine, -1.90 at the femoral neck and -1.80 at the total hip. She had no family history of osteoporosis, no relevant past medical history and had not taken any drugs which could have predisposed her to having osteoporosis. Her pregnancy was uncomplicated, but it was unclear if she breastfed her baby. She started treatment with alendronate and calcium and vitamin D supplements about 3 months after the pregnancy, but she stopped taking alendronate after a few months. Two years after the pregnancy, her T-scores were stable, with values of -3.30 at the lumbar spine, -1.80 at the femoral neck and -1.70 at the total hip. After eight years, the spine T-score was stable at -3.30 but the hip score had risen to -1.30 at the femoral neck and -1.30 at the total hip. She experienced no further fractures were identified during this period.

### **Case 3**

A 34-year-old woman suddenly developed back pain immediately after delivering her first child. She had imaging four months after the pregnancy, which revealed multiple vertebral fractures affecting T5, T6, T7, T9, T10, T11 and T12. Five months after the pregnancy, a DEXA scan showed severe spinal osteoporosis with a T-score of -4.80 at the lumbar spine and osteopenia at the hip with a T-score of -2.40 at the femoral neck and -1.70 at the total hip. She had no family history of osteoporosis and no relevant past medical history. She did not take any drugs which could have predisposed her to having osteoporosis, and her pregnancy was uncomplicated. She breastfed her baby for six months and then started taking risedronate and calcium and vitamin D supplements. She remained on this treatment for three years following which time, a repeat DEXA had showed an increase in BMD, with a T-score of -3.20 at the lumbar spine, -1.80 at the femoral neck and -1.30 at the total hip. She then was offered treatment with intravenous ZA, as she wished to have another baby. She received three intravenous infusions of ZA 5mg at yearly intervals and stopped therapy. Following completion of ZA, a repeat DEXA showed further improvement with a spine T-score of -2.90, a femoral neck T-score of -1.40 and a total hip T-score of -1.30. She had a second pregnancy eight years after her first and suffered another episode of back pain during this, but she had no new fractures. She remains stable to date, having received no further treatment.

### **Case 4**

A 35-year-old woman complained of back pain while lifting her baby two months after her first pregnancy. However, no diagnosis was made at this time. Two years after the pregnancy, an X-ray showed multiple vertebral fractures affecting T7, T8, T12, L1, L2, L3, L4 and L5, and a DEXA scan showed T-scores of -4.20 at the lumbar spine, -3.80 at the femoral neck and -3.30 at the total hip. She had no family history of osteoporosis, but she had received thromboprophylaxis with low molecular weight heparin due to a strong family history of thromboembolic disease. She also took corticosteroid inhalers for asthma during pregnancy and had been treated with a LHRT agonist seven years before the pregnancy for endometriosis. Her pregnancy was uncomplicated, and she breastfed her baby for two years. Two years after the pregnancy, she started taking calcium and vitamin D supplements. A repeat DEXA scan three years after the pregnancy showed her hip BMD had slightly improved, with T-scores of -3.60 at the femoral neck and -3.00 at the total hip. Her spine could not be scanned this time due to multiple fractures. Six years after the pregnancy, she received her first of three yearly intravenous infusions of ZA, as she was planning a further pregnancy. However, she eventually did not have another pregnancy. At the last review ten years after the initial pregnancy, her DEXA scan showed T-scores of -3.30 at the femoral neck and -2.70 at the total hip, and she had not sustained any further fractures.

### **Case 5**

A 38-year-old woman suffered an acute episode of back pain eighteen weeks after delivering her second child. Subsequently, spine imaging showed multiple vertebral fractures affecting T7, T9, T10, T11, T12 and all lumbar vertebrae. Eight months after this pregnancy, a DEXA scan revealed T-scores of -1.60 at the femoral neck and -1.20 at the total hip. Her lumbar spine could not be scanned due to her vertebral fractures. She had no family history of osteoporosis, but she had a history of low-trauma wrist and ankle fractures before pregnancy. She took enoxaparin as thromboprophylaxis during her second pregnancy due to concern about a possible deep vein thrombosis, and she also received local corticosteroid injections during her second pregnancy to manage her pre-existing chronic back pain. Her pregnancy was uncomplicated, but it is unclear if she breastfed her second baby. Nine months after the pregnancy, she started treatment with calcium and vitamin D supplements. Three years later, a DEXA scan showed stable levels of BMD with T-scores of -1.70 at the femoral neck and -1.10 at the total hip. She did not sustain further fractures during an eleven-year follow-up period.

### **Case 6**

A 37-year-old woman experienced left buttock and hip pain one week after delivering her second child. MRI and CT scans two months after her second pregnancy showed an insufficiency fracture of her left sacral ala. A DEXA scan four months after this pregnancy showed T-scores of -1.10 at the lumbar spine, +0.60 at the femoral neck and +0.60 at the total hip. She had no family history of osteoporosis. She took corticosteroid inhalers for asthma during the pregnancy but also had taken vitamin D supplements throughout her pregnancy and continued to take them postpartum. The pregnancy was uncomplicated, and she breastfed her baby for an unknown duration of time. She was diagnosed as possibly having a PAO-related fracture. Within a few months, her fracture healed, her pain decreased, and she was caring well for her second baby. She did not sustain any further fractures during four years of follow-up. No further DEXA scans have been performed.

### **Case 7**

A 37-year-old woman presented with back pain eight weeks after delivering her first child. Subsequent imaging revealed multiple vertebral fractures affecting T11, T12, L1, L2 and L3. However, a DEXA scan two years after the pregnancy showed T-scores of -1.60 at the lumbar spine, -1.80 at the femoral neck and -0.70 at the total hip. She had a family history of osteoporosis but no relevant past medical history. She did not take any drugs which could have predisposed her to having osteoporosis, and her pregnancy was uncomplicated. It is unclear if she breastfed her baby. Two years after the pregnancy, she started treatment with risedronate and calcium and vitamin D supplements. Her back pain completely subsided while on this treatment for five years. Seven years after the pregnancy, her T-scores had improved to -0.70 at the lumbar spine, -1.60 at the femoral neck and -0.40 at the total hip. Fourteen years after the pregnancy, she presented with sudden onset back pain without a history of trauma, but imaging did not show any further fractures, and her T-scores at the hip remained stable with values of -1.30 at the femoral neck and -0.80 at the total hip. Her spine BMD was not measured this time due to her multiple fractures.

### **Case 8**

A 34-year-old woman presented with ankle pain and swelling during her first pregnancy. An MRI scan two months before she delivered her first child revealed multiple ankle fractures. Three months after the pregnancy, a DEXA scan showed T-scores of -1.80 at the lumbar spine, -2.20 at the femoral neck and -1.90 at the total hip. She had no family history of osteoporosis, but she received low molecular weight heparin injections as thromboprophylaxis during pregnancy and continued taking lorazepam during pregnancy to manage pre-existing schizophrenia. Her pregnancy was uncomplicated, and she breastfed her baby for three weeks. Six months after the pregnancy, she was commenced on calcium and vitamin D supplements. Her most recent DEXA scan approximately two years after the pregnancy revealed stable T-scores with values of -1.80 at the lumbar spine, -2.20 at the femoral neck and -1.70 at the total hip. She did not sustain any more fractures during four years of follow-up, and her presenting fractures had healed.

### **Case 9**

A 29-year-old woman presented with sudden onset back pain three months after delivering her first child. An MRI scan one year after the pregnancy showed multiple vertebral fractures affecting T7, T8, T9, T10, T11, T12 and L1. She had no family history of osteoporosis and no relevant past medical history. She did not take any drugs which could have predisposed her to having osteoporosis, and her pregnancy was uncomplicated. She breastfed her baby for an unknown duration. Two years after the pregnancy, she started taking calcium and vitamin D supplements. A DEXA scan four years after the pregnancy revealed T-scores of -2.60 at the lumbar spine, -0.50 at the femoral neck and -0.50 at the total hip. She did not sustain any further fractures during five years of follow-up, including a second pregnancy which occurred four years after her first pregnancy.

### **Case 10**

A 31-year-old woman presented with back pain four months after delivering her first child. Seven months later, an X-ray showed three vertebral fractures in the lumbar spine. A DEXA scan nine months later showed T-scores of -3.50 at the lumbar spine, -1.00 at the femoral neck and -1.40 at the total hip. She had a family history of osteoporosis but no relevant past medical history. She received low molecular weight heparin as thromboprophylaxis immediately after delivery. Her pregnancy was complicated by an antepartum haemorrhage. She breastfed her baby for an unknown duration. She started taking calcium and vitamin D supplements one year after the pregnancy. Another DEXA scan two years later showed her T-scores had increased to -2.60 at the lumbar spine, -0.40 at the femoral neck and -0.90 at the total hip. However, three years later, her T-score at the lumbar spine dropped to -3.20, improved to a value of -0.20 at the femoral neck and remained relatively stable at the total hip with a T-score of -1.00. Following this scan, she commenced treatment with alendronate. She did not sustain any further fractures during four years of follow-up, including during a second pregnancy three years after her first pregnancy.

### **Case 11**

A 32-year-old woman presented with back pain at thirty weeks gestation during her first pregnancy. An MRI scan one month before delivery revealed multiple vertebral fractures affecting T9, T10, T11, T12, L1 and L2. Three months after the pregnancy, she was also found to have lost ten centimetres in height and was noted to have a thoracic kyphosis. A DEXA scan four months after delivery showed T-scores of -2.80 at the lumbar spine, -0.70 at the femoral neck and -0.80 at the total hip. She had no family history of osteoporosis, but she took clobazam and corticosteroid inhalers during pregnancy for the treatment of pre-existing epilepsy and asthma. She sustained two pulmonary embolisms before the pregnancy and was treated with the factor Xa inhibitors tinzaparin and enoxaparin as thromboprophylaxis during the pregnancy. Her pregnancy was uncomplicated, and she did not breastfeed her baby. She was prescribed calcium and vitamin D supplements eleven months after the pregnancy. Four years later, she also commenced treatment with alendronate for a period of three years. A DEXA scan eleven years later showed increased BMD, with T-scores of -2.20 at the lumbar spine, 0.00 at the femoral neck and -0.10 at the total hip. She did not sustain any further fractures during follow-up of thirteen years.

### **Case 12**

A 40-year-old woman complained of back pain while lifting her baby six weeks after her third pregnancy. Three months later, an MRI scan revealed multiple vertebral fractures affecting T12, L1, L2, L3 and L4. A subsequent DEXA scan showed T-scores of -1.40 at the femoral neck and -1.30 at the total hip. No reading could be obtained at the lumbar spine due to her fractures. She had no family history of osteoporosis and no relevant past medical history. She did not take any drugs which could have predisposed her to having osteoporosis, and the pregnancy was uncomplicated. She breastfed her baby for eight months. She was treated with calcium and vitamin D supplements four months after the pregnancy. She stopped taking calcium supplements after three years because her dietary calcium intake had increased. Three years after the pregnancy, her T-scores were -1.10 at the femoral neck and -1.10 at the total hip. However, after five years, she developed further back pain and was found to have vertebral fracture at L5. Following this, she was prescribed teriparatide. At the most recent follow-up six years after the initial presentation, a DEXA scan showed stable levels of BMD with T-scores of -1.20 at the femoral neck and -1.30 at the total hip.

### **Case 13**

A 36-year-old woman presented with back pain seven months after her first pregnancy. An X-ray one year later showed multiple vertebral fractures affecting T8, T11, T12 and L1, and a DEXA scan showed T-scores of -2.80 at the lumbar spine, -2.60 at the femoral neck and -2.40 at the total hip. She had no family history of osteoporosis. She smoked for ten years before the pregnancy, but she did not take any drugs which could have predisposed her to having osteoporosis, and her pregnancy was uncomplicated. She breastfed her baby for nine months. One year after the pregnancy, she started treatment with alendronate and calcium and vitamin D supplements. Two years later, her T-scores had remained stable with values of -2.90 at the lumbar spine, -2.70 at the femoral neck and -2.40 at the total hip. No more fractures were identified during the two-year follow-up period.

### **Case 14**

A 32-year-old woman complained of back pain after lifting her baby one month after her first pregnancy. Three months later, an MRI scan revealed multiple vertebral fractures affecting T4, T6, T7, T9, T11, T12 and L1. A subsequent DEXA scan showed T-scores of -3.60 at the lumbar spine, -2.60 at the femoral neck and -2.10 at the total hip. She had a family history of osteoporosis, and she received low molecular weight heparin as thromboprophylaxis during pregnancy due to being diagnosed with a slow cerebral blood flow. She also took corticosteroid inhalers for asthma during pregnancy, but her pregnancy was uncomplicated. She breastfed her baby for at least four months. Five months after the pregnancy, she commenced treatment with alendronate and calcium and vitamin D supplements. However, she stopped taking alendronate after one month. Two years after the pregnancy, her T-scores had improved to -2.40 at the lumbar spine, -2.40 at the femoral neck and -2.20 at the total hip. Her most recent DEXA scan four years after the pregnancy revealed T-scores of -1.90 at the lumbar spine, -2.50 at the femoral neck and -2.10 at the total hip. Although the BMD values increased and she did not sustain any further fractures, she was still in pain throughout her four-year follow-up period.

### **Case 15**

A 35-year-old woman presented with back pain six weeks after delivering her second child. An MRI scan identified multiple fractures in thoracic vertebrae T6, T7, T8, T9, T10, T11 and T12. A subsequent DEXA scan showed T-scores of -2.80 at the femoral neck and -1.70 at the total hip. No readings were obtained at the lumbar spine due to the vertebral fractures. She had a family history of osteoporosis but was prescribed low molecular weight heparin as antenatal and postnatal thromboprophylaxis due to sustaining a deep vein thrombosis when she was 19 years old. The pregnancy was complicated by hyperemesis gravidarum. She breastfed her second baby for three months, before having to stop to receive treatment with diazepam for her back pain. She had a trial of treatment on alendronate and then teriparatide but was intolerant of both drugs. About one year after the pregnancy, she commenced a course of three intravenous infusions of ZA at yearly intervals, along with vitamin D supplements. Three years later, her T-scores had improved to -2.40 at the femoral neck and -1.30 at the total hip. She sustained no further fractures during the three-year follow-up period.

### **Case 16**

A 32-year-old woman presented with back pain, five-centimetres height loss and a thoracic kyphosis two weeks before delivering her first child. A year later, an MRI scan identified multiple vertebral fractures affecting T7, T9, T11, T12, L2, L3, L4 and L5. Two years later, a DEXA scan showed T-scores of -2.10 at the femoral neck and -1.30 at the total hip. No BMD measurements could be obtained at the spine due to the vertebral fractures. She had a family history of osteoporosis but no relevant past medical history. She stopped smoking eleven years before her pregnancy, and she took levothyroxine for hypothyroidism during pregnancy. The pregnancy was complicated by hyperemesis gravidarum, and it is unclear if she breastfed her baby. She started taking calcium and vitamin D supplements one year after the pregnancy. While on this treatment, she felt well, and her pain diminished. Two years later, her height was stable, and her T-scores increased to -1.80 at the femoral neck and -1.00 at the total hip. Despite this, she commenced treatment with teriparatide. Her most recent DEXA scan three years after the pregnancy showed a stable BMD compared to her previous scan, with T-scores of -1.90 at the femoral neck and -0.80 at the total hip. She did not sustain any further fractures during three years of follow-up.

Supplementary Table 1.
